# A Case of Thrombotic Thrombocytopenic Purpura without Pathognomonic Schistocytes

**DOI:** 10.3390/clinpract11020033

**Published:** 2021-04-13

**Authors:** Kevin Yu, Min Yan

**Affiliations:** 1 Department of Molecular and Cell Biology, University of California, Berkeley, CA 94720, USA; 2 Department of Hematology and Oncology, Alta Bates Summit Medical Center, Summit Campus, Oakland, CA 94609, USA; Min.Yan@epic-care.com; 3 Medical Oncology and Hematology, Epic Care, Emeryville, CA 94608, USA

**Keywords:** thrombotic thrombocytopenic purpura, blood smear, schistocytes

## Abstract

Patients diagnosed with thrombotic thrombocytopenic purpura (TTP) typically present with microangiopathic hemolytic anemia (MAHA) and thrombocytopenia; these two clinical manifestations were often believed to be essential indicators of TTP. However, such indicators are not always present in every case. Here, we present a patient affected by TTP but showing no distinctive schistocytes on blood smear review. TTP was diagnosed through a critically low level of a disintegrin and metalloproteinase with thrombospondin type 1 motif, member 13 (ADAMTS13) activity. Awareness of such an atypical presentation of TTP is essential for timely treatment to prevent serious and even fatal outcomes for patients.

## 1. Introduction

The clinical features of thrombotic thrombocytopenic purpura (TTP) often include hemolytic anemia with abundant schistocytes, thrombocytopenia, renal injury, fever, and neurological deficiencies [1,2]. In particular, the presence of both thrombocytopenia and microangiopathic hemolytic anemia (MAHA) are regarded by many clinicians as cornerstones of TTP diagnosis. It is important to note, however, that these well-known features are not present in all cases of TTP; for example, it has been shown that schistocytes are not present or obvious in every case of TTP [3,4,5,6,7,8,9]. In addition, a lack of thrombocytopenia does not necessarily exclude diagnosis of TTP [4,5]. In the case of TTP we present herein, the characteristic schistocytes were not obvious on a blood smear, but the patient’s level of a disintegrin and metalloproteinase with thrombospondin type 1 motif, member 13 (ADAMTS13) activity was less than 10%. Treatment with therapeutic plasma exchange (TPE) led to remission of TTP. With the details presented, we hope to increase awareness that TTP may present as stroke and a lack of schistocytes does not exclude its diagnosis.

## 2. Case Report

A 47-year-old female was hospitalized for dysarthria. Her prior conditions included morbid obesity, hypertension, and dyslipidemia. She did not report past surgical history. The patient also reported that she had never engaged in tobacco usage, did not consume alcohol, and did use marijuana. She had no known allergies. Physical examination was remarkable for an obese female with mild difficulty in articulation. Workup done for cerebrovascular accident (CVA) included CT head with contrast which showed minimal subacute left occipital infarct. She did not have MRI of the brain. She had normal telemetry monitoring and normal echocardiogram and bubble study. Her complete blood count was unremarkable except for a finding of a platelet count of 50,000/μL (reference range 150,000–400,000/μL). The hematology service was not consulted on this admission. She had a normal size of the liver (14.5 cm) and spleen (12 cm) on ultrasound, normal levels of B12 (481 ng/mL, reference range 204–964 ng/mL), and normal levels of folate (9.6 ng/mL, reference range 7.2–15.4 ng/mL). In addition, a test for anti-phospholipid syndrome showed negative lupus anti-coagulant (PTT 38, reference range < 40; DRVVT 43, reference range < 45) and negative anti-cardiolipin IgG (<1.6, reference range < 20) and anticardiolipin IgM (2.2, reference range < 20). Thrombophilia tests showed normal levels of protein C activity (89%, reference range 70–130%), protein S activity (92%, reference range 55–123%), and anti-thrombin III activity (87%, reference range 80–120%). Factor V Leiden and prothrombin G20210A were not detected. She was subsequently started on aspirin/dipyridamole for CVA and discharged for outpatient follow-up with neurology and hematology, which unfortunately was not accomplished.

**Figure 1:**
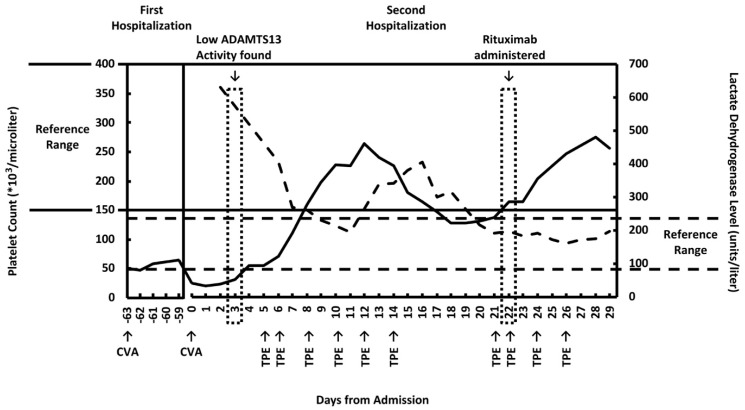
Platelet count and lactate dehydrogenase (LDH) levels over the course of hospitalization. Solid line: platelet count. Dotted line: lactate dehydrogenase levels. CVA = cerebrovascular accident. TPE = therapeutic plasma exchange. Dates of TPE and rituximab administration, known CVA, and discovery of low ADAMTS13 activity are marked. Platelet counts from the patient’s first hospitalization, which was between 63 and 59 days before her second admission, are prior to the diagnosis of thrombotic thrombocytopenic purpura (TTP). Days 0 through 29 refer to the patient’s second hospitalization, in which TTP was diagnosed and treated.

**Figure 2:**
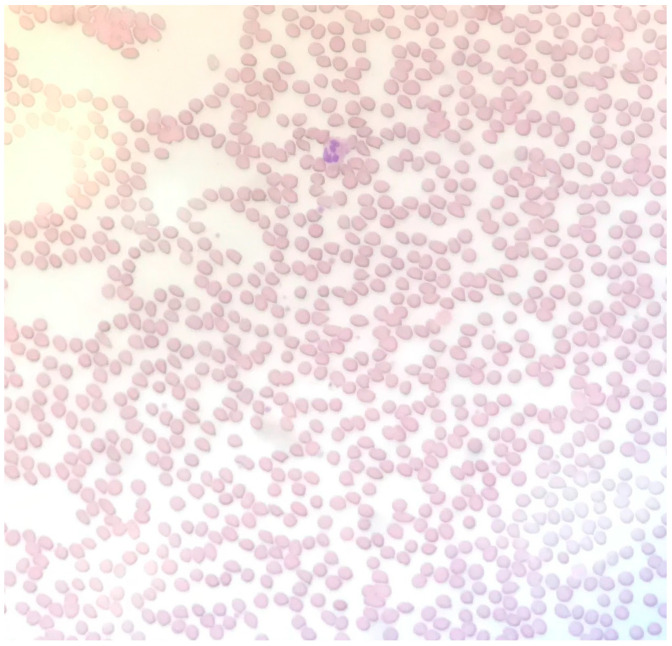
Peripheral smear showing no signs of pathognomonic schistocytes.

Two months later, the patient presented with aphasia and was re-admitted. The patient reported that she had been compliant with aspirin/dipyridamole as prescribed. MRI during this admission revealed that she had acute to subacute infarct of the left cingulate gyrus and left frontal lobe periphery zone. She was again noted to have thrombocytopenia. Her platelet counts are presented in Figure 1. Her hemoglobin level was low at admission (9.8 g/dL, reference range 11.7–15.5 g/dL) and did not change significantly throughout the course of her hospitalization. At admission, her urea nitrogen level was 19 mg/dL (reference range 6–25 mg/dL) and creatinine level was 1.41 mg/dL (reference range 0.4–1 mg/dL). She had an elevated lactate dehydrogenase (LDH) level of 629 μ/L (reference range 81–234 μ/L, Figure 1). At this admission, the hematology service was consulted. The presence of thrombocytopenia and stroke in a young patient raised suspicion of thrombotic thrombocytopenic purpura (TTP). However, the peripheral smear did not show schistocytes (Figure 2). In addition, her reticulocyte absolute count, of 0.09 M/μL (reference range 0.02–1.00 M/μL), was not elevated. Total bilirubin was 0.5 mg/dL (reference range < 1.1 mg/dL) and direct bilirubin was 0.1 mg/dL (reference range < 0.3 mg/dL). Plasma ADAMTS13 activity was less than 1% (reference range 40–130%) and an ADAMTS13 inhibitor test was reported as equivocal.

TPE therapy was administered from 5 days after admission. After 10 sessions of TPE between days 5 and 26, and one dose of intravenous rituximab on day 22 (Figure 1), the patient was discharged on day 29 with complete recovery of her symptoms and normal platelet count and LDH level.

## 3. Discussion

Our patient presented several symptoms and signs of TTP, such as thrombocytopenia, anemia with elevated LDH, and neurological deficit; however, there were no pathognomonic schistocytes on her blood smear. Her extremely low ADAMTS13 activity (<1%) confirms the diagnosis of TTP. Although her ADAMTS13 inhibitor test result was reported as equivocal, it does not exclude the diagnosis of acquired TTP. In some patients of acquired TTP, the inhibitor level is too low to be detected in the conventional mixing test for inhibitors [10]. In fact, her episode of exacerbation during the course of TPE suggests that she had acquired TTP rather than congenital TTP. Patients of congenital TTP do not have inhibitors. Hence, they respond readily to fresh frozen plasma, with the platelet count increasing to the normal range for at least two weeks after a two-unit infusion of fresh frozen plasma [11].

Previously, a pentad of thrombocytopenia, MAHA, neurological deficits, fever, and renal dysfunction was proposed to constitute the diagnosis of TTP. Subsequently a diagnosis of TTP was presumed when a patient presented with the dyad of MAHA and thrombocytopenia, based on the need to promptly treat such patients [11]. With the discovery that thrombosis of TTP is due to profound ADAMTS13 deficiency [11], it is increasingly recognized that a lack of MAHA and/or thrombocytopenia does not exclude the diagnosis of TTP, as has been described in various reports in the literature [3,4,5,6,7,8,9]. In 2002, O’Brien et al. [3] reported two patients who presented with neurological deficits with roughly normal hematological findings. They were initially treated for transient ischemic attack (TIA) or conversion disorder before they developed thrombocytopenia and MAHA two and six weeks later, respectively. Of note, ADAMTS13 activity and inhibitor levels were not tested in those two patients; hence the diagnosis of TTP should only be considered presumptive. In 2003, Tsai et al. [4] reported a patient with relapsed TTP who presented with CVA but only developed schistocytes and thrombocytopenia three weeks after the stroke. After achieving hematologic remission with plasma exchange therapy, the patient continued to experience recurrent episodes of dizziness and blurred vision. Her ADAMTS13 activity was repeatedly 10% or less and an ADAMTS13 inhibitor test was positive despite multiple normal hemoglobin levels and platelet counts. Her ADAMTS13 level increased and her symptoms subsided after rituximab therapy [4]. Similarly, two cases of relapsed TTP presenting with CVA without overt signs of MAHA and thrombocytopenia were reported by Downes et al. [5] in 2004. Four more cases of TTP presenting with symptoms suggestive of macrovascular arterial involvement and minimal schistocytes were reported in 2012 [6]. In more recent years, several cases were reported [7,8,9] in which TTP either first presented or relapsed as CVA without clear signs of MAHA and/or thrombocytopenia. Notably, it has been almost seventeen years since publication of the earliest reports of such atypical presentations of TTP [3,4]. Atypical presentation of TTP has also been incorporated into a hematology textbook [11]. However, there may still be an under-recognition of TTP presenting without typical features of MAHA or thrombocytopenia. TTP occurs at an estimated rate of 14.5 cases per 10^6^ person-years [12] and it is even rarer to have an atypical manifestation of TTP as CVA without features of MAHA, which poses a diagnostic challenge, particularly in community healthcare settings.

In regard to the etiology of a lack of overt hematological abnormalities in the uncommon cases of TTP, one might hypothesize that the extent of thrombus formation is not widespread enough to lead to fragmented red blood cells and/or thrombocytopenia. However, symptoms of TIA or stroke may occur if the early microvascular thrombosis of TTP happens to affect a vital region of the brain. Macrovascular stroke ensues if the microvascular thrombosis affects the vasa vasorum of a large artery, resulting in endothelial injury and thrombosis of the affected vessel [11]. If TPE is not started, it is plausible that patients without initial hematological abnormalities of TTP will eventually develop overt MAHA and thrombocytopenia.

Therefore, diagnosis of TTP does not require the presence of thrombocytopenia and MAHA. TTP should be included in the differential diagnosis for a patient presenting with stroke at a young age, without conventional cardiovascular risk factors, or with unexplained thrombocytopenia and/or anemia. For such patients, ADAMTS13 activity and inhibitor tests should be performed to exclude or confirm the diagnosis of TTP.

## 4. Conclusions

The case described in this report shows that TTP may indeed present as stroke without pathognomonic schistocytes. Awareness of such an atypical presentation of TTP may help prevent delay in its diagnosis. Prompt treatment may avoid catastrophic outcomes for such patients.

## Data Availability

Not applicable.

## References

[1] Tsai H.M. (2010). Pathophysiology of thrombotic thrombocytopenic purpura. Int. J. Hematol..

[2] Sadler J.E. (2017). Pathophysiology of thrombotic thrombocytopenic purpura. Blood.

[3] O’Brien T.E., Crum E.D. (2002). Atypical Presentations of Thrombotic Thrombocytopenic Purpura. Int. J. Hematol..

[4] Tsai H.M., Shulman K. (2003). Rituximab induces remission of cerebral ischemia caused by thrombotic thrombocytopenic purpura. Eur. J. Haematol..

[5] Downes K.A., Yomtovian R., Tsai H.M., Silver B., Rutherford C., Sarode R. (2004). Relapsed Thrombotic Thrombocytopenic Purpura Presenting as an Acute Cerebrovascular Accident. J. Clin. Apher..

[6] Imanirad I., Rajasekhar A., Zumberg M. (2012). A Case Series of Atypical Presentations of Thrombotic Thrombocytopenic Purpura. J. Clin. Apher..

[7] Kalish Y., Rottenstreich A., Rund D., Hochberg-Klein S. (2016). Atypical presentations of thrombotic thrombocytopenic purpura: A diagnostic role for ADAMTS13. J. Thromb. Thrombolysis.

[8] Badugu P., Idowu M. (2019). Atypical Thrombotic Thrombocytopenic Purpura Presenting as Stroke. Case Rep. Hematol..

[9] Singh B., Chan K.H., Kaur P., Modi V., Maroules M. (2020). Atypical Presentation of Thrombotic Thrombocytopenic Purpura without Hematological Features. Int. J. Hematol. Oncol. Stem Cell Res..

[10] Tsai H.M., Lian E.C. (1998). Antibodies to von Willebrand factor-cleaving protease in acute thrombotic thrombocytopenic purpura. N. Engl. J. Med..

[11] Tsai H.M., Greer J.P., Arber D.A., Glader B.E., List A.F., Means R.M., Rodgers G.M. (2019). Chapter 49: Thrombotic Thrombocytopenic Purpura, Hemolytic-Uremic Syndrome, and Related Disorders. Wintrobe’s Clinical Hematology.

[12] Tsai H.M., Rodgers G.M. (2015). Chapter 6: Acquired thrombotic thrombocytopenic purpura-a disease due to inhibitors of ADAMTS13. ADAMTS13-Biology and Disease.

